# Children education for sustainable control of liver fluke infections

**DOI:** 10.1186/s40249-022-01041-4

**Published:** 2022-12-08

**Authors:** Men-Bao Qian, Xiao-Nong Zhou

**Affiliations:** 1 National Institute of Parasitic Diseases, Chinese Center for Disease Control and Prevention (Chinese Center for Tropical Diseases Research), Shanghai, China; 2 NHC Key Laboratory of Parasite and Vector Biology, Shanghai, China; 3 WHO Collaborating Center for Tropical Diseases, Shanghai, China; 4 National Center for International Research on Tropical Diseases, Shanghai, China; 5 School of Global Health, Chinese Center for Tropical Diseases Research, Shanghai Jiao Tong University School of Medicine, Shanghai, China

**Keywords:** Liver fluke, Education, Children, Cartoon

## Abstract

Significant disease burden is caused by infections with human liver flukes, including *Clonorchis sinensis*, *Opisthorchis viverrini* and *O. felineus*. Epidemiology is characterized by high burden in the male than the female, and in the elder than the younger. Chemotherapy is the mainstream for morbidity control, but it could not prevent re-infection. Meanwhile, behavioral change on the dietary habit of ingesting raw freshwater fish is challenging. In this opinion, we argue why it is important to educate children for sustainable control of liver fluke infections. Then, the design, development and key messages of an educational cartoon for preventing liver fluke infections is introduced. Technical focuses are emphasized based on a pilot using the education cartoon for preventing clonorchiasis in China. Finally, how to further develop and verify this education strategy is discussed.

**Graphical Abstract:**

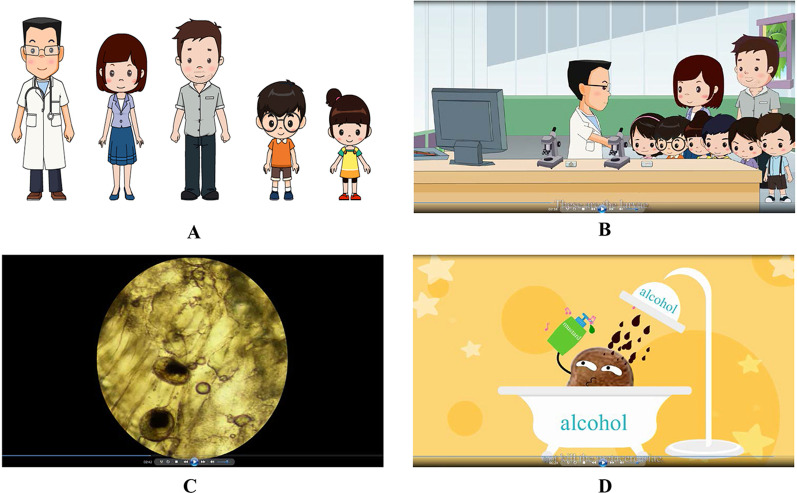

**Supplementary Information:**

The online version contains supplementary material available at 10.1186/s40249-022-01041-4.

## Background

*Clonorchis sinensis*, *Opisthorchis viverrini* and *O. felineus* are important human liver flukes, causing clonorchiasis and opisthorchiasis in human beings [1, 2]. Their life cycle involves in intermediate hosts—freshwater snails and fish, and definitive hosts—humans and vertebrate animals. Human beings are infected through the ingestion of raw or undercooked freshwater fish containing the infective larvae (metacercariae). Metacercariae enter human bodies and develop into adult worms, parasitizing in liver and biliary system and laying eggs there. Human liver flukes cause diverse symptoms and complications [3–5]. In early infection, symptoms are mild and nonspecific, of which abdominal pain and diarrhea are frequent. Persistent infection without treatment and management leads to severe complications, of which gallstone and cholecystitis are most frequent. Additionally, both *C. sinensis* and *O. viverrini* are definite carcinogen, leading to fatal sequelae—cholangiocarcinoma [6]. These liver fluke infections are predominantly prevalent in Asia and east part of Europe. An estimation of 15 million people are afflicted by *C. sinensis* in China, the Republic of Korea, northern Vietnam and part of Russia, 12 million cases by *O. viverrini* in Southeast Asia including Thailand, Laos, Cambodia, southern Vietnam and Myanmar, and another 1.6 million by *O. felineus* in Russia, Ukraine and Kazakhstan [1, 7, 8].

## Importance of education for children

Infection with human liver flukes are characterized by high prevalence and infection intensity in the male compared to the female and in adults than children, which is attributable to differential dietary habit in ingesting raw freshwater fish [9]. Severe morbidity is subsequently presented in adults especially men [10]. Thus, chemotherapy with drugs (usually praziquantel) is nowadays employed as mainstream. This strategy is high effective in term of the decrease in both prevalence and infection intensity after several rounds’ administration of drugs [11]. However, single chemotherapy strategy could not prevent re-infection, and thus prevalence and infection intensity bounce quickly when intervention stops [12]. Education is advocated to be integrated to promote behavioral change in ingestion of raw freshwater fish. However, adults especially men are highly indulged into this dietary habit, and thus education on them is challenging [13]. Indeed, compared to children, adults especially men have more knowledge on human liver flukes [13]. However, the knowledge could not effectively help them increase the awareness and subsequently abandon the ingestion of raw freshwater fish.

Besides many other factors, the mutual impact in ingesting raw freshwater fish among family members is of crucial importance, which is called familial assimilation [14]. In particular, children gradually get accustomed to raw-eating practice due to intergenerational assimilation, namely behavioral imitation from their parents. Following their growing up, the habit in ingesting raw freshwater fish is gradually established. When children grow up into adults, get married and have their own babies, the raw-eating practice will be transmitted to next generation. This theory could explain the epidemiological profiles of human liver fluke infections characterized by high prevalence in men [9]. This could also explain why liver fluke infections are endemic in east Asia for a long time. Indeed, the epidemiology of clonorchiasis in China is similar to that one century ago: similar distribution areas, similar profiles in gender and ages, and similar preparation process of raw freshwater fish dishes [15]. Additionally, the paradox between knowledge and behavior, namely more knowledge on liver flukes and more practice in ingestion of raw freshwater fish in men, is also understandable [13]. During the establishment in ingesting raw freshwater fish, children also get some knowledge from external environments. However, such unsystematic knowledge could not benefit the increase in awareness of the importance to prevent liver fluke infections and establishment of the belief not to ingest raw freshwater fish in future.

## Development of an educational cartoon for children

Based on a national evaluation on educational products for helminthiases in China, educational products focusing on prevention of liver fluke infections were inadequate [16]. Considering the characteristics of children, educational video will be a choice to deliver knowledge and sharp proper behavior, which has been employed in other helminthiases [17]. The accessibility to multimedia is also feasible, which reached 98.4% in primary and middle schools in 2020 in China [18]. Thus, an educational cartoon was developed for preventing liver fluke infections (Additional file 1). The length of this cartoon is about seven minutes, which avoids distraction of students’ attentions in a longer video. The cartoon is narrative based on a story. In brief, two students didn’t come to the class at the beginning of new semester. The teacher was told they got illness because of abdominal pain and diarrhea. The teacher and several classmates came to hospital to visit them. Then, through dialogue and demonstration, a doctor introduced basic knowledge on liver flukes (Box 1). In this story, 10 key messages are delivered. The key messages were repeatedly presented through the plot to impress the audience.

**Fig. 1:**
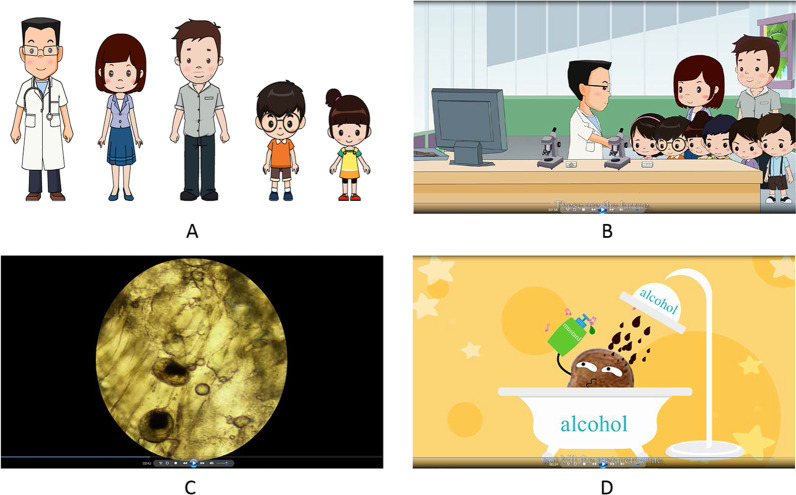
Selected illustration from the educational cartoon, “a changing life of liver fluke”. **A** Major characters, including doctor, teacher, father and two students. **B** Introduction of liver flukes by the doctor. **C** The demonstration of larvae (metacercariae) in freshwater fish under microscope. **D** An impersonated plot to indicate the uselessness in killing metacercariae through drinking alcohol and consuming food condiments

Several technical points deserve to be emphasized (Fig. 1). First, it is difficult to introduce the life cycle of liver flukes, while it is also challenging to deliver the knowledge if life cycle is not demonstrated. Thus, the life cycle was vividly presented through computer and microscopy, in which many real pictures were included to increase the impression. Thus, the introduction of life cycle becomes interesting. Second, to further increase the interesting, several humorous dialogues were introduced. The doctor said the morphology of adult flukes looks like sunflower seed. Then, one student asked whether the infected students ate the flukes directly, which promoted the doctor to explain the transmission route. Furthermore, one student astonished and asked whether the doctor operated to detect the flukes, which paved the way to introduce the diagnosis method. Additionally, metacercariae bathed in alcohol, which emphasizes that liver flukes are resistant to alcohol and other food condiments. Third, because diverse morphology of liver flukes was presented, the cartoon is named “a changing life of liver fluke” to draw attentions.

## Box 1: Key messages delivered in an educational cartoon—“a changing life of liver fluke”

**Table:** 

1) What is liver fluke? Physical pictures and animation demonstration to show the morphology.
2) How is it transmitted? Ingestion of raw or undercooked freshwater fish.
3) What is the harm? Three levels. Frequent symptoms (abdominal pain and diarrhea) at early infection stage, chronic complications (gallstone and cholecystitis) due to persistent infection and carcinogenicity (liver cancer) at final stage.
4) How to diagnose? Detection of eggs in feces.
5) How to treat? Taking drugs.
6) How to prevent? Don’t ingest raw or undercooked freshwater fish/Cooking freshwater fish completely.
7) What are misunderstood? a) **False**: There exists transmission between humans. **Truth**: No direct transmission between humans. This piece of knowledge could avoid the potential discrimination on the individual ingesting raw freshwater fish. b) **False**: There exists little harm to adults. **Truth**: More damage in adults because of persistent infection. c) **False**: Metacercariae could be killed by alcohol and food condiments. **Truth**: Only complete cooking kills metacercariae.
8) What needs to be done? Don’t ingest raw or undercooked freshwater fish and persuade parents not to do it.

## How to educate children for preventing liver fluke infections

A cluster-controlled trial was implemented in primary schools in China to explore the control strategy against clonorchiasis based on the educational cartoon [19, 20]. Compared to baseline, in interventional school after education, the percentage of students with knowledge on transmission route, early symptoms, complications and carcinogenicity all increased significantly, while the proportion of students ingesting raw freshwater fish during the year immediately before the survey decreased significantly. The percentage of students with the belief not to ingest raw freshwater fish in future increased obviously. In control group, the knowledge increased in a less degree compared to the change in interventional school, while practice and belief didn’t change significantly.

The cartoon was integrated into diverse educational activities. Educational classes were set specially. At the beginning, trained teacher discussed with the students about the knowledge on liver flukes and practice in ingesting raw freshwater fish. Key points were asked. Then, the educational cartoon was broadcasted. Then, the teacher negotiated with the students again about the key points. The cartoon was then broadcasted again. To impress the students, educational brochure with information extracted from the cartoon was developed and distributed to each student. Then, drawing and essay writing competitions on the morphology of liver flukes and the story on preventing liver fluke infections were implemented, in which excellent pieces were awarded. Additionally, the educational cartoon was broadcasted periodically.

## Conclusion

In term of high health burden caused by liver fluke infections, high re-infection in single chemotherapy and challenge in behavioral change in adults, and dominance of familial assimilation in establishment of the dietary habit of ingesting raw freshwater fish in childhood, it is argued here to implement education on children for sustainable control of liver fluke infections. This educational strategy with the cartoon was proved preliminarily to be effective in pilot, which is also being applied in some clonorchiasis-endemic areas in China. It is expected to further test and evaluate this strategy. First, the sustainability should be further assessed through longer time observation as well as in higher grade students. Second, intergenerational assimilation promotes the transmission of raw-eating practice from parents to children. It is interesting to explore the feasibility of reverse action, namely the behavioral change of parents by children through education. Indeed, it is emphasized that children should persuade their parents to abandon ingesting raw freshwater fish in this cartoon. Third, the epidemiology and transmission determinants of three species of liver flukes are similar, which increases the potential application in combat against both clonorchiasis and opisthorchiasis. It is welcomed to adjust and translate the cartoon into other languages, and subsequently evaluate and apply for preventing both clonorchiasis and opisthorchiasis in other endemic countries. Finally, although single chemotherapy in adults is not sustainable, it could control morbidity rapidly. Thus, the integration of children education, chemotherapy in adults, as well as other measures could probably increase the effectiveness, which also needs to be explored.

## Supplementary Information

**Additional file 1. **Video of the educational cartoon. 

## Data Availability

Not applicable.

## References

[1] Qian MB, Utzinger J, Keiser J, Zhou XN, Clonorchiasis (2016). Lancet.

[2] Harrington D, Lamberton PHL, McGregor A (2017). Human liver flukes. Lancet Gastroenterol Hepatol.

[3] Choi MS, Choi D, Choi MH, Ji Z, Li Z, Cho SY (2005). Correlation between sonographic findings and infection intensity in clonorchiasis. Am J Trop Med Hyg.

[4] Qiao T, Ma RH, Luo XB, Luo ZL, Zheng PM (2012). Cholecystolithiasis is associated with *Clonorchis sinensis* infection. PLoS ONE.

[5] Qian MB, Li HM, Jiang ZH, Yang YC, Lu MF, Wei K (2021). Severe hepatobiliary morbidity is associated with *Clonorchis sinensis* infection: the evidence from a cross-sectional community study. PLoS Negl Trop Dis.

[6] Bouvard V, Baan R, Straif K, Grosse Y, Secretan B, El Ghissassi F (2009). A review of human carcinogens–Part B: biological agents. Lancet Oncol.

[7] WHO (1995). Control of foodborne trematode infections. Report of a WHO Study Group. World Health Organ Tech Rep Ser.

[8] Zhao TT, Feng YJ, Doanh PN, Sayasone S, Khieu V, Nithikathkul C (2021). Model-based spatial-temporal mapping of opisthorchiasis in endemic countries of Southeast Asia. elife.

[9] Zhu TJ, Chen YD, Qian MB, Zhu HH, Huang JL, Zhou CH (2020). Surveillance of clonorchiasis in China in 2016. Acta Trop.

[10] Qian MB, Chen YD, Fang YY, Xu LQ, Zhu TJ, Tan T (2011). Disability weight of *Clonorchis sinensis* infection: captured from community study and model simulation. PLoS Negl Trop Dis.

[11] Choi MH, Park SK, Li Z, Ji Z, Yu G, Feng Z (2010). Effect of control strategies on prevalence, incidence and re-infection of clonorchiasis in endemic areas of China. PLoS Negl Trop Dis.

[12] Li Z, Xin H, Qian MB, Sun J, Yang Y, Chen Y (2022). *Clonorchis sinensis* reinfection rate and reinfection determinants: a prospective cohort study in Hengxian county, Guangxi, China. J Infect Dis.

[13] Qian MB, Zhou CH, Jiang ZH, Yang YC, Lu MF, Wei K (2022). Epidemiology and determinants of *Clonorchis sinensis* infection: a community-based study in southeastern China. Acta Trop.

[14] Qian MB, Jiang ZH, Zhou CH, Ge T, Wang X, Zhou XN (2020). Familial assimilation in transmission of raw-freshwater fish-eating practice leading to clonorchiasis. PLoS Negl Trop Dis.

[15] Oldt F (1927). Is *Clonorchis* a health menace in China?. Chin Med J.

[16] Qian MB, Zhou CH, Zhu HH, Zhu TJ, Huang JL, Chen YD (2019). Assessment of health education products aimed at controlling and preventing helminthiases in China. Infect Dis Poverty.

[17] Bieri FA, Gray DJ, Raso G, Li YS, McManus DP (2012). A systematic review of preventive health educational videos targeting infectious diseases in schoolchildren. Am J Trop Med Hyg.

[18] Digital China Development Report. (2020). https://doi.org/http://www.gov.cn/xinwen/2021-07/03/content_5622668.htm. Accessed 27 Jul 2022 (In Chinese).

[19] Qian MB, Jiang ZH, Gan XQ, Zhao JG, Li W, Zheng WJ (2019). Effect of health education on the awareness and control of clonorchiasis in primary school students. Chin J Parasitol Parasit Dis.

[20] Qian MB, Gan XQ, Zhao JG, Zheng WJ, Li W, Jiang ZH (2020). Effectiveness of health education in improving knowledge, practice and belief related to clonorchiasis in children. Acta Trop.

